# Selective Cross-Subject Transfer Learning Based on Riemannian Tangent Space for Motor Imagery Brain-Computer Interface

**DOI:** 10.3389/fnins.2021.779231

**Published:** 2021-11-03

**Authors:** Yilu Xu, Xin Huang, Quan Lan

**Affiliations:** ^1^School of Software, Jiangxi Agricultural University, Nanchang, China; ^2^Software College, Jiangxi Normal University, Nanchang, China; ^3^Department of Neurology, First Affiliated Hospital of Xiamen University, Xiamen, China

**Keywords:** transfer learning, cross-subject, source selection, Riemannian tangent space, motor imagery

## Abstract

A motor imagery (MI) brain-computer interface (BCI) plays an important role in the neurological rehabilitation training for stroke patients. Electroencephalogram (EEG)-based MI BCI has high temporal resolution, which is convenient for real-time BCI control. Therefore, we focus on EEG-based MI BCI in this paper. The identification of MI EEG signals is always quite challenging. Due to high inter-session/subject variability, each subject should spend long and tedious calibration time in collecting amounts of labeled samples for a subject-specific model. To cope with this problem, we present a supervised selective cross-subject transfer learning (sSCSTL) approach which simultaneously makes use of the labeled samples from target and source subjects based on Riemannian tangent space. Since the covariance matrices representing the multi-channel EEG signals belong to the smooth Riemannian manifold, we perform the Riemannian alignment to make the covariance matrices from different subjects close to each other. Then, all aligned covariance matrices are converted into the Riemannian tangent space features to train a classifier in the Euclidean space. To investigate the role of unlabeled samples, we further propose semi-supervised and unsupervised versions which utilize the total samples and unlabeled samples from target subject, respectively. Sequential forward floating search (SFFS) method is executed for source selection. All our proposed algorithms transfer the labeled samples from most suitable source subjects into the feature space of target subject. Experimental results on two publicly available MI datasets demonstrated that our algorithms outperformed several state-of-the-art algorithms using small number of the labeled samples from target subject, especially for good target subjects.

## Introduction

A motor imagery (MI) brain-computer interface (BCI) has drawn great attention for decades, since it can help a subject directly manipulate an electronic equipment using his brain activity evoked by imagined movements, without the participation of the traditional muscle-dependent pathway (He et al., 2015). It can not only help the stroke patients recover their neurological disorders, but also give able-bodied people a novel way to control an external device (Wolpaw et al., 2002). Therefore, it plays an important role in rehabilitation engineering, military, and entertainment, etc.

Non-invasive electroencephalogram (EEG) is a popular recording modality in MI BCI due to its high safety and high temporal resolution, which is extremely crucial for the application of real-time BCI. However, MI EEG data analysis is quite challenging. The reason is that EEG signals are inherently weak, non-stationary, and easily contaminated by interference and noise (Sample et al., 2019). Moreover, compared with other traditional EEGs, such as event-related potentials (ERP) and steady-state visual evoked potentials (SSVEP), MI EEG signals have less obvious features, because they are evoked by spontaneous movement imagination without external stimulus, whereas ERP and SSVEP are invoked by some external stimulation (Blankertz et al., 2011; Yin et al., 2015). Consequently, MI EEG signals have higher inter-session/subject variability and fewer categories of BCI tasks than ERP and SSVEP. In MI BCI, each subject needs a tedious and annoying calibration time for a subject-specific classifier before performing real-time BCI tasks. A retraining session always increases user frustration.

Deep learning is a promising machine learning technique, which has been widely used in natural language processing and computer vision and so on Huang et al. (2019, 2020). However, it cannot be directly applied to the small training set scenario.

Transfer learning (TL) and semi-supervised learning (SSL) have been tried to reduce the need of abundant labeled samples. TL transfers the labeled samples from different source domains into the target domain. A domain denotes a subject, a session, a task, or a device. SSL simultaneously utilizes the labeled and unlabeled samples from the same subject. Both TL and SSL can shorten the calibration effort for the target subject by utilizing the samples as much as possible.

Here, we pay more attention to TL since it can use more samples than SSL. Generally, TL can be categorized into three groups, including instance TL (ITL), feature-representation transfer (FRT), and parameter TL (PTL) (Pan and Yang, 2010). Instance TL aims to transfer parts of the data in the source domains by reweighting. FRT tries to transfer a good feature representation for the target domain. PTL successively transfers and updates some parameters of the model under the assumption that the source and target domains share these parameters.

Among these groups, ITL approaches are most popular since they are easy to implement. Specifically, they can be divided into two categories.

The first category is filter based ITL, which aims to reweight the labeled sets from different domains based on the filters. In MI BCI, the common spatial patterns (CSP) approach is only effective when the labeled samples from a subject are abundant (Ramoser et al., 2000). Recently, regularized CSP (RCSP) approaches, belonging to filter based ITL, have been designed for the small training set scenario. In the framework of RCSP, the filtered EEG samples from the source and target subjects are separately reweighted based on their similarities which are always measured using different metrics, such as kullback-leibler (KL) divergence, Frobenius norm, cosine distance and so on (Kang et al., 2009; Cheng et al., 2017; Xu et al., 2019). A RCSP based on dynamic time warping (DTW-RCSP) approach aligned the labeled samples from all source subjects to the average of a few target samples from the same class (Azab et al., 2020). In the module of feature extraction, RCSP approaches can generate more reliable CSP spatial filters for the target subject by effectively utilizing the labeled samples from the source subjects. However, calculating the optimal regularization terms might impose computational burden.

The second category is data alignment based ITL. On past decade, Riemannian alignment- (RA-) based ITL approaches have drawn a growing attention in EEG-based BCI since affine transformation can make the covariance matrices from different domains similar. Zanini et al. (2018) proposed a RA-based ITL approach to center the covariance matrices from each domain with respect to their reference matrix. Such reference matrix is the Riemannian mean of the covariance matrices of some resting trials in the corresponding domain. All re-centered covariance matrices from different source domains are concatenated altogether to train a minimum distance to mean (MDM) classifier based on Riemannian Gaussian distributions. Rodrigues et al. (2019) proposed a Riemannian procrustes analysis (RPA) approach using the steps of re-centering, stretching, and rotation for the covariance matrices from different domains to minimize the differences between the source and target domains as much as possible. Zhang and Wu (2020) presented a cross-subject manifold embedded knowledge transfer (MEKT) approach to boost zero-training for the target subject by combining the labeled samples from the source subjects with the unlabeled samples from the target subject. MEKT first performs RA to generate aligned covariance matrices for all subjects and then converts all aligned matrices into the tangent space feature vectors. Finally, MEKT finds optimal projection matrices for the tangent space feature vectors from the source and target subjects to reduce the joint probability distribution shift between subjects. Since most classifiers are designed for the Euclidean space instead of the Riemannian manifold, Euclidean alignment- (EA-) based approaches extend RA-based approaches in the Euclidean space using the Euclidean mean as the reference matrix (He and Wu, 2020). Both RA and EA can shorten the differences between the covariance matrices from the source and target subjects.

Inspired by MEKT, we focus on cross-subject TL based on Riemannian tangent space. In the calibration phase, labeled samples from target subject are successively processed by the modules of signal preprocessing, feature extraction, and classification to build a subject-specific classifier. To reduce the calibration time, the labeled samples from different source subjects can be transferred into the same modules for a more robust classifier.

Nevertheless, most cross-subject TL approaches utilize the labeled samples from all source subjects. Lotte and Guan (2011) designed a RCSP framework which evaluated the differences between the whole CSP feature set from all source subjects and the CSP feature set from the target subject, and then added two different weights for these two sets. Zhang and Wu (2020) found two optimal projection matrices for the whole tangent space vector set from all source subjects and the unlabeled tangent space vector set from the target subject to lower their dimensions and differences.

To promote the positive transfer, the appropriate source subjects should be selected. Zanini et al. (2018) transferred all good source subjects (those with a precision higher than 0.75) for the target subject. To select suitable source subjects, Liang and Ma (2020) temporarily assigned the labeled samples from the target subject as the testing set, and the labeled samples from the candidate source subject as the training set. Then, the source subjects with higher classification accuracy were selected. Zhang and Wu (2020) presented a domain transferability estimation (DTE) approach based on MEKT, which selected the source subjects with the highest transferability.

In this paper, we first propose a supervised selective cross-subject TL (supervised SCSTL, sSCSTL) approach based on Riemannian tangent space for MI classification, which collects only a few labeled samples from target subject and transfers the labeled samples from most suitable source subjects. Moreover, we present an unsupervised SCSTL (uSCSTL) approach and a semi-supervised SCSTL (ssSCSTL) approach to investigate the role of the unlabeled samples from target subject. A sequential forward floating search (SFFS) method (Pudil et al., 1994) is used to iteratively select suitable source subjects for all SCSTL approaches.

The contribution of this paper lies in two aspects. Firstly, we iteratively select appropriate source subjects, instead of simply selecting them based on their classification performance, transferability and so on. Secondly, we consider SCSTL using different versions to exploit the labeled and unlabeled samples from target subject.

The remainder of this paper is structured as follows. In Section “Methods,” our SCSTL approaches are described in detail. In Section “Experimental Results,” the effectiveness of our approaches is validated by some experimental results. A discussion of results is presented in Section “Discussion.” Finally, our conclusions are drawn in Section “Conclusion.”

## Methods

**FIGURE 1:**
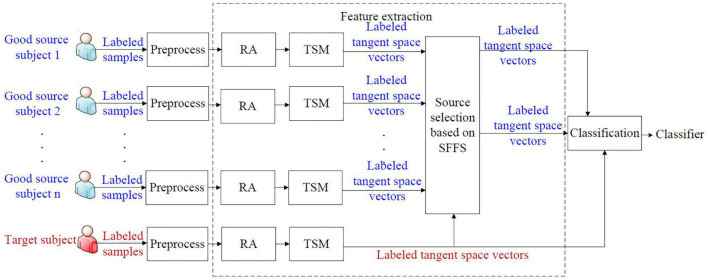
The framework of supervised selective cross-subject transfer learning (sSCSTL).

We propose a supervised selective cross-subject transfer learning (sSCSTL) approach which utilizes the labeled samples from the selected good source subjects and the target subject simultaneously. In our opinion, the source subjects with good classification performance are more eligible to be selected than those with bad performance, since the labeled samples from the bad source subjects might mess up the feature distribution of the target subject. The framework of sSCSTL is shown in Figure 1.

In Figure 1, the labeled samples from each subject are band-pass filtered in the signal preprocessing module. During the feature extraction phase, the filtered labeled samples from each subject are first converted into the labeled covariance matrices. To preliminarily shorten the differences between subjects, the labeled covariance matrices from each subject are transformed into the aligned labeled covariance matrices by performing RA. To train a classifier in the Euclidean space, the aligned labeled matrices from each subject are converted into corresponding labeled tangent space vectors by executing tangent space mapping (TSM). To obtain best source selection, based on the SFFS method, the labeled tangent space vectors from different good source subjects are selected into the selected tangent space vector set by comparing with the labeled tangent space vector set from the target subject. Finally, the labeled tangent space vectors from the most suitable good source subjects and the target subject are fed into the classification module to obtain a subject-specific classifier.

Next, we introduce the sSCSTL approach in detail.

### Riemannian Alignment

In MI BCI, supervised CSP is the most popular feature extraction algorithm for single user. Covariance matrices from different classes are used to learn optimal spatial filters which can maximize the variance differences between two classes. Such spatial filters are used to extract low-dimensional features for the labeled and unlabeled samples. If the labeled samples are noisy, and/or few, covariance matrices might generate unreliable spatial filters. In this fashion, covariance matrices are processed in the Euclidean space. However, they belong to a smooth Riemannian manifold of symmetric positive definite (SPD) matrices, instead of Euclidean space. Thus, in this paper, we first handle covariance matrices in the differentiable Riemannian manifold.

All SPD matrices can form a Riemannian manifold. Here are the basic concepts of SPD matrix and its manifold. Let *P_i_* and *P_j_* be the *i*th SPD matrix and the *j*th SPD matrix, respectively, where *P*_*i*_, *P*_*j*_ ∈ *ℝ*^*N*_*c*_ × *N*_*c*_^ and *N_c_* is the number of channels. Their manifold is *N*_*c*_ × (*N*_*c*_ + 1)/2 dimensional. *P_i_* and *P_j_* can be regarded as the points of the manifold. The Riemannian distance *δ*(*P*_*i*_, *P*_*j*_) between *P_i_* and *P_j_* is the length of the minimum curve connecting them. It can be calculated by Moakher (2005).


(1) δ(Pi,Pj)=||log(Pi-1Pj)||F=[∑k=1Nclog2λk]1/2

where || ⋅ ||_F_ denotes the Frobenius norm, ${\left\{\lambda_{k}\right\}}_{k=1}^{N_{c}}$ are the eigenvalues of Pi-1Pj. There exist important properties for the Riemannian distance as follows:


$$\delta \left(P_{i},P_{j}\right)=\delta \left(P_{j},P_{i}\right)$$

δ(Pi,Pj)=δ(Pi-1,Pj-1)

$$\delta \left(P_{i},P_{j}\right)=\delta \left(W^{\text{T}}P_{i}W,W^{\text{T}}P_{j}W\right),\forall W\epsilon \text{Gl}\left(N_{c}\right)$$

The third property is crucial for the context of signal processing. It is named congruence invariance which means that the distance between the two SPD matrices is invariant after affine transformation using an invertible matrix. Suppose that Gl(*N*_*c*_) is the set of all *N*_*c*_ × *N*_*c*_ invertible matrices belonging to the space of square real matrices. The center of all SPD matrices is generally used for affine transformation, which can be calculated using Riemannian distance.

The Riemannian mean *M_R_*, namely geometric mean, is the center point of the manifold which can be calculated using Riemannian distance as follows:


(2) $$M_{R}=\underset{M}{\text{argmin}}\sum_{\text{k}=1}^{N}\delta^{2}\left(P_{k},M\right),$$

where *N* denotes the number of all SPD matrices on the manifold. There is no closed-form way to calculate the Riemannian mean. An iterative method can be used to effectively obtain the Riemannian mean (Fletcher and Joshi, 2004).

We can perform affine transformation using the center of all SPD matrices as the reference matrix. RA executes the following transformation using the Riemannian mean *M_R_* as the reference matrix:


(3) P´i=MR-1/2PiMR-1/2.

Likewise, EA extends RA using the Euclidean mean *M_E_* as the reference matrix (He and Wu, 2020). The Euclidean mean *M_E_*, namely arithmetic mean, is defined as the center point which can minimize the sum of squared Euclidean distances between other matrices and the center point as below:


(4) $$M_{E}=\underset{M}{\text{argmin}}\sum_{\text{k}=1}^{N}d^{2}\left(P_{k},M\right)=\frac{1}{N}\sum_{\text{k}=1}^{N}P_{k}.$$

where *d*(⋅, ⋅) computes the Euclidean distance between the two SPD matrices. Since the Euclidean mean can be easily computed, it is widely used so far.

In this paper, we perform RA for all covariance matrices from each subject since RA is more suitable for the Riemannian manifold.

For RA-based ITL approaches, all aligned covariance matrices from each domain are centered at the identity matrix after affine transformation using each domain’s Riemannian mean as the reference matrix. This property makes the aligned covariance matrices from different domains comparable, which can be testified by the following (Zhang and Wu, 2020).


ℳ(MR-1/2P1MR-1/2,MR-1/2P2MR-1/2,⋯,MR-1/2PNMR-1/2)

=MR-1/2ℳ(P1,P2,⋯,PN)MR-1/2

(5) =MR-1/2MRMR-1/2=I

where ℳ(*P*_1_, *P*_2_, ⋯ , *P*_*N*_) is the Riemannian mean operation, and *I* is an identity matrix.

Moreover, each aligned covariance matrix is nearly whitened since it is approximately an identity matrix. More details can be seen in Zhang and Wu (2020). Consequently, RA can preliminarily reduce the inter-domain differences.

### Tangent Space Mapping

After RA, the aligned covariance matrices are usually input to an MDM classifier (Zanini et al., 2018; Rodrigues et al., 2019). However, most classifiers, such as linear discriminant analysis (LDA) and support vector machine (SVM), are designed for the Euclidean space. Thus, we perform tangent space mapping (TSM) for all aligned matrices from each subject.

**FIGURE 2:**
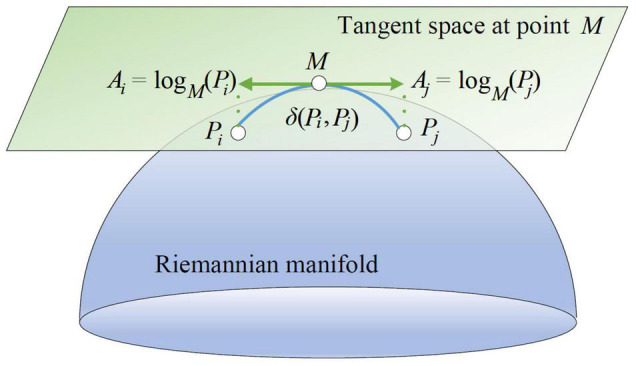
A Riemannian manifold and its tangent space at a point.

As mentioned above, all SPD matrices lie in a differentiable Riemannian manifold. Then their derivatives at a matrix on the manifold form a tangent space. The tangent space has the same dimensions as the manifold. A Riemannian manifold and its tangent space at a point can be illustrated in Figure 2.

As depicted in Figure 2, *P_i_* and *P_j_* belong to the Riemannian manifold. On the tangent space at point *M*, *A_i_* and *A_j_* are the derivatives of *P_i_* and *P_j_*, respectively. *A_i_* can be regarded as the logarithmic mapping of *P_i_* at *M* as below:


(6) $$A_{i}=\log_{M}\left(P_{i}\right)=M^{1/2}\log\left(M^{-1/2}P_{i}M^{-1/2}\right)M^{1/2}.$$

Conversely, the exponential mapping of *A_i_* at *M* is the SPD matrix *P_i_* defined as:


(7) $$P_{i}={\text{exp}}_{M}\left(A_{i}\right)=M^{1/2}\exp\left(M^{-1/2}{\text{A}}_{i}M^{-1/2}\right)M^{1/2}.$$

Riemannian distance can be also defined as:


$$\delta \left(M,P_{i}\right)={||\log_{M}\left(P_{i}\right)||}_{M}={||A_{i}||}_{M}$$

$$={||\text{vec}\left(M^{-1/2}A_{i}M^{-1/2}\right)||}_{2}={||\text{vec}\left(\log\left(M^{-1/2}P_{i}M^{-1/2}\right)\right)||}_{2}$$

(8) $$={||\text{vec}\left({\overset{´}{A}}_{i}\right)||}_{2}={||{\text{a}}_{i}||}_{2},$$

where ||*A*_*i*_||_*M*_ is the norm of *A_i_* on the tangent space at point *M*, and $\text{vec}\left({\overset{´}{A}}_{i}\right)$ vectorizes a symmetry matrix ${\overset{´}{A}}_{i}$. Let ${\overset{´}{A}}_{i}$ and a_*i*_ be *log*(*M*^−1/2^*P*_*i*_*M*^−1/2^) and $\text{vec}\left({\overset{´}{A}}_{i}\right)$, respectively. Without loss of generality, we apply a $\sqrt{2}$ coefficient for out-of-diagonal elements of ${\overset{´}{A}}_{i}$ and transform the upper triangular part of modified ${\overset{´}{A}}_{i}$ into a *N*_*c*_ × (*N*_*c*_ + 1)/2 column vector a_*i*_ as below:


(9) $${\text{a}}_{i}=\text{vec}\left({\overset{´}{A}}_{i}\right)=[{\overset{´}{A}}_{i_{1,1}};\sqrt{2}{\overset{´}{A}}_{i_{1,2}};{\overset{´}{A}}_{i_{2,2}};\sqrt{2}{\overset{´}{A}}_{i_{1,3}};\sqrt{2}{\overset{´}{A}}_{i_{2,3}};{\overset{´}{A}}_{i_{3,3}};\cdots ;{\overset{´}{A}}_{i_{N_{c},N_{c}}}]$$

where A´i∈j,kA´i. Then, a_*i*_ is the tangent space vector of the SPD matrix *P_i_* at point *M*.

Furthermore, there is an approximation in terms of distance between the manifold and its tangent space as below (Tuzel et al., 2008):


(10) $$\delta \left(P_{i},P_{j}\right)\approx {||{\text{a}}_{i}-{\text{a}}_{j}||}_{2}$$

where *P_i_* and *P_j_* are locally distributed into the manifold. Their tangent space vectors at point *M* are a_*i*_ and a_*j*_, respectively. Such approximation stands only when *M* is the Riemannian mean of the manifold.

Therefore, Riemannian tangent space is Euclidean and locally homomorphic to the Riemannian manifold (Barachant et al., 2013). In the TSM phase of our proposed sSCSTL, all aligned covariance matrices {MR-1/2PiMR-1/2}i=1N from each domain are converted into corresponding tangent space vectors {ai=vec(log(MR-1/2PiMR-1/2))}i=1N using *M_R_* as the reference matrix.

### Source Selection Based on Sequential Forward Floating Search

As mentioned above, due to high inter-subject variability and expensive computational burden, it is unsuitable to utilize the features from all source subjects. In this paper, we only transfer the labeled tangent space vectors from most suitable good source subjects.

We define the transferability for the selected tangent space vector set *Sel* to predict its usefulness for the tangent space vector set 𝒟_*T*_ from the target subject as in Zhang and Wu (2020):


(11) transferability(Sel,𝒟T)=||SbSel||1||SbSel,𝒟T||1,

where SbSel is the between-class scatter matrix of *Sel*. Similarly, SbSel,𝒟T is the scatter matrix between *Sel* and 𝒟_*T*_. Then, ||SbSel||1 is used for the discriminability of between-class of *Sel*, and ||SbSel,𝒟T||1 is used to evaluate the differences between the selected source subjects and the target subject.

Instead of simply integrating the labeled tangent space vector sets from all source subjects, the labeled tangent space vector set from the good source subject is iteratively selected to add into or remove from the selected tangent space vector set to maximize its transferability. Our proposed source selection procedure for sSCSTL uses the framework of SFFS to obtain convergence of selection. More details are shown in **Algorithm 1**.

**Algorithm 1**: The source selection procedure for sSCSTL
**Input:** *N*_*TL*_ labeled tangent space vectors from the target subject ${𝒟}_{TL}={\left\{{\text{a}}_{i}^{\left(TL\right)},y_{i}^{\left(TL\right)}\right\}}_{i=1}^{N_{TL}}$, *N*_*S_k_*_ labeled tangent space vectors from the *k*th good source subject ${𝒟}_{S_{k}}={\left\{{\text{a}}_{i}^{\left(S_{k}\right)},y_{i}^{\left(S_{k}\right)}\right\}}_{i=1}^{N_{S_{k}}}$, *k* = {1, 2, ⋯ , *N*_*GS*_}, where *N*_*GS*_ is the number of good source subjects. Let $y_{i}^{\left(TL\right)}$ and $y_{i}^{\left(S_{k}\right)}$ be the labels of tangent space vectors from target and source subjects, respectively.
**Output:** The finally selected tangent space vector set *Sel*;
**Initialize:** The initially selected tangent space vector set *Sel*_0_ = ∅ and its transferability *Trans*_0_ = 0, the initially remaining tangent space vector set $Rem_{0}={\left\{{𝒟}_{S_{k}}\right\}}_{k=1}^{N_{GS}}$, *n=1*;
**Repeat**
  **Step 1:** The labeled tangent space vector set from the most suitable good source subject is selected and added into *Sel*_*n*−1_

$${𝒟}_{S_{good}}=\underset{{𝒟}_{S_{k}}\in Rem_{n-1}}{\text{argmax}}\left(transferability\left(Sel_{n-1}+{𝒟}_{S_{k}},{𝒟}_{TL}\right)\right);$$

$$Sel_{n}=Sel_{n-1}+{𝒟}_{S_{good}};Rem_{n}=Rem_{n-1}-{𝒟}_{S_{good}};$$

$$Trans_{n}=transferability\left(Sel_{n},{𝒟}_{TL}\right);$$

n=n+1;

**Step 2:** The labeled tangent space vector set from the most unsuitable good source subject is selected and removed from *Sel*_*n*_
  **if** *n* > 2 **then**

$${𝒟}_{S_{bad}}=\underset{{𝒟}_{S_{k}}\in Sel_{n}}{\text{argmax}}\left(transferability\left(Sel_{n}-{𝒟}_{S_{k}},{𝒟}_{TL}\right)\right);$$

**if** *transferability*(*Sel*_*n*_ − 𝒟_*S*_*bad*__, 𝒟_*TL*_) > *Trans*_*n*−1_ **then**

$$Sel_{n-1}=Sel_{n}-{𝒟}_{S_{bad}};Rem_{n-1}=Rem_{n}+{𝒟}_{S_{bad}};$$

$$Trans_{n-1}=transferability\left(Sel_{n-1},{𝒟}_{TL}\right);$$

n=n-1;

gotoStep 2;

**else** go to Step 1;
**end**
**end**
  **Until** *n* = *N*_*GS*_

$$Sel=Sel_{arg\max_{k}\left(Trans_{k}\right)}.$$

For sSCSTL, labeled covariance matrices from each subject are converted into labeled tangent space vectors using their Riemannian mean as the reference matrix.

In **Algorithm 1**, during each iteration, *Sel*_*n*_ and *Trans*_*n*_ separately denote the currently selected tangent space vector set from *n* good source subjects and its transferability, whereas *Rem*_*n*_ denotes the currently remaining tangent space vector set from (*N*_*GS*_ − *n*) good source subjects. After all iterations, the selected tangent space vector set with the highest transferability is chosen as the finally selected tangent space vector set *Sel*.

Since ||SbSel,𝒟T||1 in Equation (11) is evaluated without using the labels of *Sel* and 𝒟_*T*_, we modify the source selection procedure in **Algorithm 1** for an unsupervised SCSTL (uSCSTL) approach and a semi-supervised SCSTL (ssSCSTL) approach to investigate the role of unlabeled samples from the target subject.

For uSCSTL, unlabeled covariance matrices from target subject are converted into corresponding tangent space vectors 𝒟_*TU*_ using their Riemannian mean as the reference matrix. Then, in each iteration, the currently selected tangent space vector set *Sel*_*n*_ is compared with 𝒟_*TU*_ to obtain its transferability (*transferability*(*Sel*_*n*_, *D*_*TU*_)).

For ssSCSTL, all covariance matrices from target subject are projected into corresponding tangent space vectors (𝒟_*T*_ = 𝒟_*TL*_ + 𝒟_*TU*_) using their Riemannian mean as the reference point. Then, more tangent space vectors from target subject can be used to yield more convincing transferability (*transferability*(*Sel*_*n*_, *D*_*T*_)).

### Classification

The supervised LDA classifier has been widely used because of simplicity and rapid computational speed, which is crucial for real time MI BCI. Nevertheless, it is of poor quality for high dimensional features, such as the tangent space vectors. Therefore, we use a supervised shrinkage LDA (sLDA) classifier (Peck and Van Ness, 1982) which not only inherits the advantages of LDA but also behaves well in high dimensions.

Our goal of sSCSTL, uSCSTL, and ssSCSTL is to extract discriminative features for the classifier. In our classification module, no unlabeled tangent space vectors from the target subject are utilized for the classifier. Therefore, for sSCSTL and ssSCSTL, the labeled tangent space vectors from the selected good source subjects and target subject are fed into the sLDA classifier after the best source selection based on SFFS, whereas for uSCSTL, only the labeled tangent space vectors from the selected good source subjects are used for sLDA.

## Experimental Results

### Datasets

In this paper, the MI dataset from 52 healthy subjects (Cho et al., 2017) and the BCI competition IV dataset 1 (Tangermann et al., 2012) were used to evaluate the effectiveness of our proposed methods in MI classification.

(1) MI dataset from 52 healthy subjects (MI1): 64-channel MI EEG signals from 52 healthy subjects were recorded at a sampling rate of 512 Hz. Each subject executed the assigned MI task when a random instruction (“left hand” or “right hand”) appeared on the screen for 3 s. Only a single session was performed for each subject, which consisted of five or six runs. In each run, the MI experiments per class were repeated 20 times. Consequently, there were a total of 200 or 240 trials per subject. This dataset was much suitable for cross-subject TL due to the existence of many subjects.
(2) BCI competition IV dataset 1 (MI2): this dataset was comprised of the calibration data and the evaluation data. However, we only chose the calibration data for our experiments since the evaluation data contained the periods in which the subject had no control intention. For the calibration data, 59-channel EEG signals from seven healthy subjects (a, b, c, d, e, f, and g) were collected at a sampling rate of 100 Hz. Each subject was shown a visual cue for 4 s and performed the cued MI task (“left hand” or “foot”). In total, for each subject, 100 trials per class were gathered. All EEG signals were band-pass filtered between 0.05 Hz and 200 Hz.

### Electroencephalogram Data Preprocessing

For dataset MI1, the BBCI toolbox (Blankertz et al., 2016) was used for preprocessing. All EEG signals from each subject were preprocessed by common average reference, spectrally filtered by a third order Butterworth filter with cutoff frequencies of 8 and 30 Hz, and temporally segmented from 0.5 to 2.5 s after the instruction onsets.

For dataset MI2, all EEG recordings from each subject were band-pass filtered between 8 and 30 Hz using a fiftieth order finite impulse response filter. Then, the filtered EEG recordings were extracted from the time interval between 0.5 and 3.5 s after the visual cue onsets.

**FIGURE 3:**
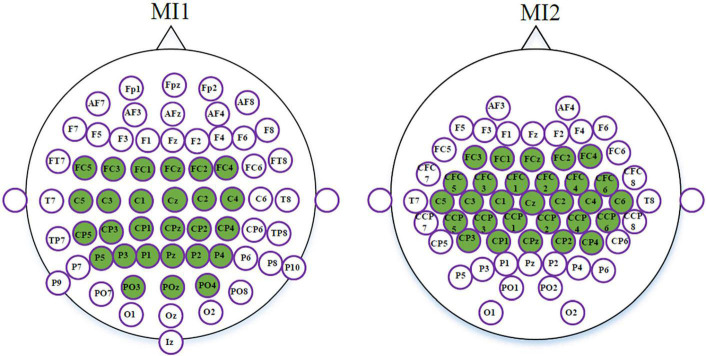
The selected channels for MI1 and MI2.

As mentioned above, the dimensionality of tangent space vectors closely relates to the number of channels. High-dimensional tangent space vectors may lead to curse of dimensionality. Therefore, for datasets MI1 and MI2, 27 and 29 channels were separately selected from the sensorimotor areas. In Figure 3, the selected channels used for datasets MI1 and MI2 are marked in green.

### Experimental Design

First, we selected the good subjects as the source subjects based on their high classification accuracies. In our experiments, the labels of their samples were assumed to be known already. All their samples were used for cross-subject TL. If one of the good subjects was the target subject, the remaining good subjects were his source subjects. All samples from each target subject were randomly partitioned into the labeled set for training and the unlabeled set for testing. To avoid randomness involved, this process was repeated ten times and the average accuracies of the unlabeled set were reported. The ratio of number of labeled samples to all samples (*R_l_*) ranged from 10 to 40% with step 10%. Correspondingly, the ratio of number of unlabeled samples to all samples (*R_u_*) varied from 90 to 60%.

### Baseline Algorithms

We compared our proposed algorithms (sSCSTL, ssSCSTL, and uSCSTL) with the following baseline algorithms. They can be grouped into three categories according to their feature spaces:

(1) Euclidean space algorithms:
  (a) CSP (Ramoser et al., 2000): it was a traditional feature extraction algorithm for MI. We used LDA as its classifier.
  (b) CSP with generic learning regularization (GLRCSP) (Lu et al., 2009): it learned generic learning regularized CSP filters to realize cross-subject TL. LDA was its classifier.
  (c) Composite CSP (CCSP) (Kang et al., 2009): it weighed covariance matrices from different subjects using the KL divergence measure based on the framework of RCSP. LDA was also its classifier.
(2) Riemannian space algorithm: all covariance matrices belong to the Riemannian manifold. We designed a covariance matrix -based feature extraction algorithm, named COV, which directly input all labeled covariance matrices into the MDM classifier without RA.
(3) Riemannian Tangent space algorithms:
  (a) Tangent space (TS) feature extraction algorithm (Barachant et al., 2012): it successively performed RA and TSM for all covariance matrices. Then, the tangent space vectors were fed into the LDA classifier.
  (b) Manifold embedded knowledge transfer (MEKT) (Zhang and Wu, 2020): it combined the labeled tangent space vectors from the source subjects with the unlabeled tangent space vectors from the target subject. sLDA was used as its classifier.
  (c) Domain transferability estimation (DTE) (Zhang and Wu, 2020): it identified one most suitable source subject for the target subject based on MEKT. sLDA was also used for MI classification.

We set the hyper-parameters of all baseline algorithms instructed by their publications. Our proposed algorithms and seven baselines can also be further divided into two groups. The first group consists of CSP, GLRCSP, CCSP, COV, TS, sSCSTL, and ssSCSTL. They are all supervised or semi-supervised feature extraction algorithms. The second group includes MEKT, DTE, and uSCSTL. They are unsupervised feature extraction algorithms.

### Classification Accuracies

#### Classification Performance on Dataset MI1

For a small percentage of subjects, their classification accuracies hardly reach the benchmark of 70%. This phenomenon has been named BCI illiteracy. It was suggested that at least 40 labeled samples per class can reduce BCI illiteracy (Blankertz et al., 2007). Therefore, for dataset MI1, we chose the good subjects whose accuracies were over 80% on average when 40 or 48 labeled samples per class (*R*_*l*_ = 40%) were randomly selected for 10 times and trained by CSP and LDA.

In our experiments, we focused on the good and bad subjects to discuss how they influenced the BCI performance. Furthermore, we cared about the supervised, semi-supervised, and unsupervised algorithms to investigate the role of the labeled and unlabeled samples.

The classification performances of supervised and semi-supervised algorithms for good subjects on dataset MI1 are given in Table 1 using their 40 or 48 labeled samples per class (*R_l_* = 40%). Then, the performances of unsupervised algorithms for the same good subjects are shown in Table 2 using their remaining unlabeled samples (*R*_*u*_ = 60%). Meanwhile, the means and standard deviations (Std) of good subjects for two groups of algorithms are listed in Tables 1, 2, respectively.

**TABLE 1:** Average classification accuracies of supervised and semi-supervised algorithms for good subjects on dataset MI1 (*R_l_* = 40%).

	**3**	**4**	**14**	**23**	**41**	**43**	**48**	**Mean (Std)**
CSP	89.50	82.50	96.92	81.33	83.67	96.92	82.33	87.60 (6.90)
GLRCSP	89.58	82.92	96.17	80.17	85.83	95.92	80.58	87.31 (6.78)
CCSP	90.08	84.33	96.92	80.75	85.50	96.08	82.08	87.96 (6.54)
COV	86.42	77.33	96.92	73.08	73.92	93.17	75.58	82.35 (9.78)
TS	90.17	77.92	96.25	80.83	80.50	96.08	82.83	86.37 (7.70)
sSCSTL	92.00	95.75	100.00	94.25	97.67	99.83	97.75	96.75 (2.94)
ssSCSTL	91.33	99.42	100.00	99.42	99.42	100.00	100.00	98.51 (3.18)
								

**TABLE 2:** Average classification accuracies of unsupervised algorithms for good subjects on dataset MI1 (*R*_*u*_ = 60%).

	**3**	**4**	**14**	**23**	**41**	**43**	**48**	**Mean (Std)**
MEKT	90.42	84.17	95.25	82.17	86.92	94.83	82.75	88.07 (5.51)
DTE	89.42	80.75	95.00	78.58	82.42	92.33	81.92	85.77 (6.38)
uSCSTL	88.83	97.17	99.50	93.83	97.50	100.00	99.17	96.57 (3.99)

As shown in Table 1, there were only seven good subjects selected from 52 subjects on dataset MI1 according to the performance of CSP. For all Euclidean space-based algorithms, there were no obvious differences among them. CSP slightly outperformed GLRCSP and lagged behind CCSP a little. Without RA, the Riemannian space-based algorithm COV performed the worst. For Riemannian tangent space-based algorithms, the mean of TS was lower than that of CSP. sSCSTL showed its superiority among all supervised algorithms. A paired *t*-test showed that the result of sSCSTL was statistically higher than that of CSP (*p* = 0.0067), GLRCSP (*p* = 0.0055), CCSP (*p* = 0.0064), COV (*p* = 0.0052), and TS (*p* = 0.0077). ssSCSTL stood out itself with the help of unlabeled samples. In Table 2, all unsupervised algorithms utilized the labeled samples from the selected source subjects and added the remaining unlabeled samples from the good target subject. MEKT surpassed most of supervised algorithms in Table 1, except for sSCSTL. Although DTE had the same framework of MEKT, it performed worse than MEKT. The possible reason was that there was only one suitable source subject selected. For uSCSTL, the average classification accuracies of six good subjects were up to 90%.

**FIGURE 4:**
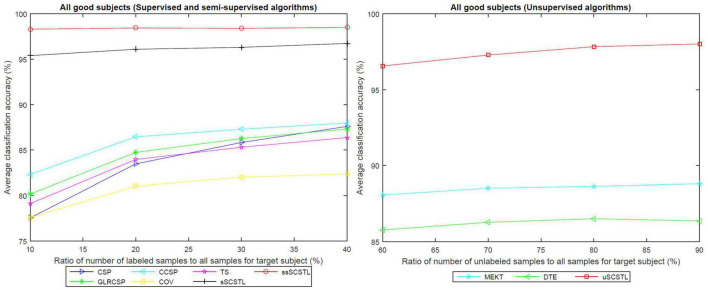
The means of accuracies of all good subjects on dataset MI1 for different algorithms with varying *R_l_* or *R_u_*.

To further evaluate the effect of the labeled and unlabeled samples from the target subject, we plotted the means of accuracies of all good subjects on dataset MI1 for different algorithms with varying *R_l_* or *R_u_* in Figure 4.

As depicted in Figure 4, all supervised and semi-supervised algorithms provided good classification performances with varying *R_l_* from 10 to 40%. Except for COV, most algorithms outperformed CSP when few labeled samples from target subject were available. Moreover, the classification performances of most algorithms improved obviously along with the increasement of *R_l_*. However, sSCSTL and ssSCSTL steadily showed compelling validity. In addition, the curves of unsupervised algorithms improved slowly with the increasement of *R_u_*, suggesting that the labeled samples were more useful for the performance than the unlabeled samples. Nevertheless, even using 60% unlabeled samples from the target subject, the average classification accuracy was over 95% for uSCSTL.

Then, since there were 45 bad subjects on dataset MI1, we did not provide their average classification accuracies one by one. Instead, in Tables 3, 4, we separately investigated how many bad subjects were trapped in BCI illiteracy with varying *R_l_* and *R_u_*. Here, those whose average classification accuracies were less than 70% were regarded as unqualified subjects.

**TABLE 3:** The number of unqualified subjects whose accuracies were less than 70% on dataset MI1 for supervised and semi-supervised algorithms with varying *R_l_*.

	**10%**	**20%**	**30%**	**40%**	**Mean**
CSP	45	42	37	32	39
GLRCSP	44	39	36	31	37.5
CCSP	42	37	33	31	35.75
COV	43	43	43	41	42.5
TS	43	42	40	38	40.75
sSCSTL	35	34	33	34	34
ssSCSTL	34	34	34	31	33.25
					

**TABLE 4:** The number of unqualified subjects whose accuracies were less than 70% on dataset MI1 for unsupervised algorithms with varying *R*_*u*_.

	**60%**	**70%**	**80%**	**90%**	**Mean**
MEKT	45	45	45	45	45
DTE	45	45	45	45	45
uSCSTL	38	37	38	37	37.5

As shown in Table 3, Euclidean space-based algorithms sharply reduced the number of unqualified subjects when their labeled samples increased. It implied that they performed well when abundant labeled samples from target subject were available. In contrast, other algorithms reduced BCI illiteracy slowly with the increasement of *R_l_*, suggesting that their performances did not excessively rely on the number of labeled samples. sSCSTL reduced more unqualified subjects than CCSP when *R_l_* was 10%. In Table 4, the number of unqualified subjects barely changed with the increasement of *R_u_* for all unsupervised algorithms. uSCSTL also reduced more unqualified subjects than MEKT when *R_u_* was 60%.

**FIGURE 5:**
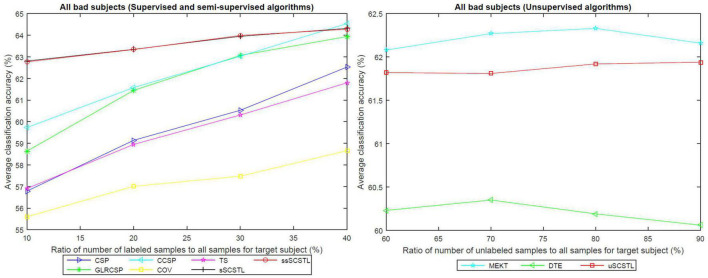
The means of accuracies of all bad subjects on dataset MI1 for different algorithms with varying *R_l_* or *R_u_*.

Like Figure 4, the means of accuracies of all bad subjects on dataset MI1 for different algorithms with varying *R_l_* or *R_u_* are plotted in Figure 5.

As illustrated in Figures 4, 5, the advantages of sSCSTL and ssSCSTL for all bad subjects were less apparent than those for good ones. In Figure 5, there were few differences between sSCSTL and ssSCSTL. Possible explanation was that low class-discriminability of unlabeled samples from bad target subjects influenced the performance improvements of ssSCSTL. In addition, CCSP and GLRCSP still outperformed CSP, TS, and COV. Additionally, the average classification accuracies provided by uSCSTL were slightly lower than those of MEKT with varying *R_u_*. The performance of DTE showed the importance of using source subjects as much as possible.

#### Classification Performance on Dataset MI2

On dataset MI2, five good subjects (a, d, e, f, and g) were chosen out of the seven subjects depending on the same rule as mentioned above. Then subjects b and c were regarded as bad subjects. The classification performances of supervised and semi-supervised algorithms for all subjects on dataset MI2 are shown in Table 5 using their 40 labeled samples per class (*R_l_* = 40%). And the performances of unsupervised algorithms for the same subjects are given in Table 6 using their remaining unlabeled samples (*R*_*u*_ = 60%).

**TABLE 5:** Average classification accuracies of supervised and semi-supervised algorithms for all subjects on dataset MI2 (*R_l_* = 40%).

	**a**	**b**	**c**	**d**	**e**	**f**	**g**	**Mean (Std)**
CSP	87.67	69.33	68.75	87.17	92.50	89.50	91.17	83.73 (10.20)
GLRCSP	86.17	73.33	72.08	84.08	93.75	89.92	89.92	84.18 (8.42)
CCSP	87.67	74.50	71.17	86.75	93.00	90.75	90.92	84.96 (8.60)
COV	81.42	65.50	67.75	76.00	74.50	80.00	76.58	74.54 (5.93)
TS	88.33	65.50	70.83	82.42	89.83	89.33	92.33	82.65 (10.46)
sSCSTL	79.33	69.92	68.08	99.92	100.00	98.17	100.00	87.92 (14.90)
ssSCSTL	83.25	67.50	71.50	100.00	100.00	100.00	100.00	88.89 (14.64)
								

**TABLE 6:** Average classification accuracies of unsupervised algorithms for all subjects on dataset MI2 (*R*_*u*_ = 60%).

	**a**	**b**	**c**	**d**	**e**	**f**	**g**	**Mean (Std)**
MEKT	78.33	73.50	73.00	70.00	90.33	80.83	91.92	79.70 (8.59)
DTE	71.00	72.25	71.25	81.08	86.33	68.42	79.33	75.67 (6.61)
uSCSTL	63.00	65.58	62.67	100.00	100.00	99.75	99.92	84.42 (19.35)

As shown in Table 5, among all Euclidean space-based algorithms, GLRCSP and CCSP outperformed CSP on average. COV still performed the worst due to without RA. For Riemannian tangent space-based algorithms, the average accuracy of TS was slightly lower than that of CSP. sSCSTL and ssSCSTL stood out themselves on average. However, for bad subjects, GLRCSP and CCSP were comparably superior to other algorithms. For most good subjects (d, e, f, and g), sSCSTL and ssSCSTL exhibited their superiorities. Paired *t*-test results between sSCSTL and other supervised algorithms showed that the performance improvements of sSCSTL over others were statistically significant, such as sSCSTL vs. CSP (*p* = 0.1795), sSCSTL vs. GLRCSP (*p* = 0.2901), sSCSTL vs. CCSP (*p* = 0.3753), sSCSTL vs. COV (*p* = 0.0260), and sSCSTL vs. TS (*p* = 0.1631). As reported in Table 6, although uSCSTL showed its compelling validity on average, MEKT performed better than uSCSTL for bad subjects.

**FIGURE 6:**
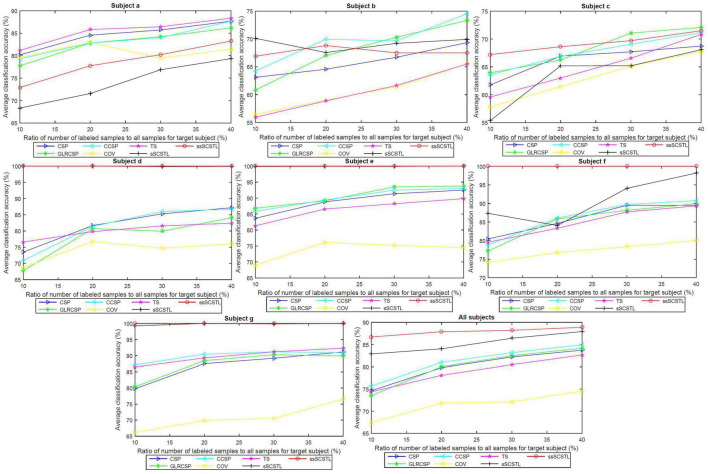
Average classification accuracies of all subjects on dataset MI2, and their means, with varying *R_l_*, for supervised and semi-supervised algorithms.

**FIGURE 7:**
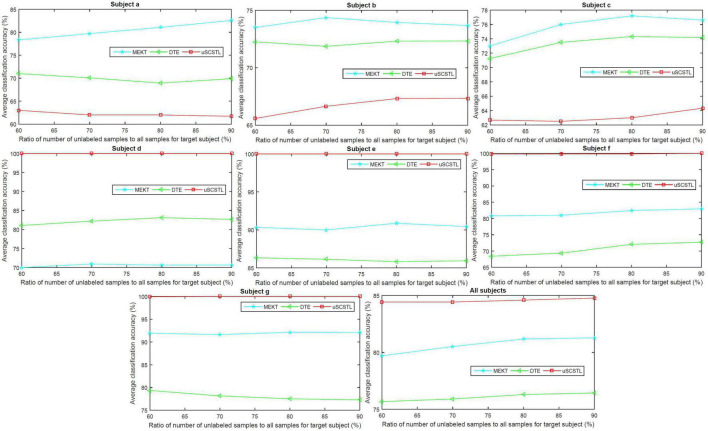
Average classification accuracies of all subjects on dataset MI2, and their means, with varying *R_u_*, for unsupervised algorithms.

To further evaluate the role of labeled and unlabeled samples, for different algorithms, we plotted the average classification accuracies of all subjects on dataset MI2, and their means, with varying *R_l_* and *R_u_* in Figures 6, 7, respectively.

In the last subfigure of Figure 6, ssSCSTL outperformed other algorithms on average with the help of unlabeled samples from target subject. The gap between sSCSTL and ssSCSTL became small with the increasement of labeled samples from target subject. For Euclidean space-based algorithms, CCSP and GLRCSP still performed better than CSP on average. Riemannian tangent space-based algorithm TS showed higher performance than Riemannian space-based algorithm COV.

Moreover, as depicted in other subfigures of Figure 6, sSCSTL and ssSCSTL showed amazing accuracies for most good subjects (d, e, f, and g), suggesting that the available samples from a good subject can provide positive cross-subject TL. Exceptionally, a good subject (a) benefited little from TL algorithms. Additionally, sSCSTL and ssSCSTL performed more unstably than CCSP and GLRCSP for bad subjects (b and c) in total.

As shown in Figure 7, the average classification accuracies of uSCSTL were inferior to those of MEKT and DTE for the good subject (a) and bad subjects (b and c). However, uSCSTL stood out itself for other subjects. Therefore, in the last subfigure of Figure 7, uSCSTL performed better than other unsupervised algorithms overall.

### Data Visualization

**FIGURE 8:**
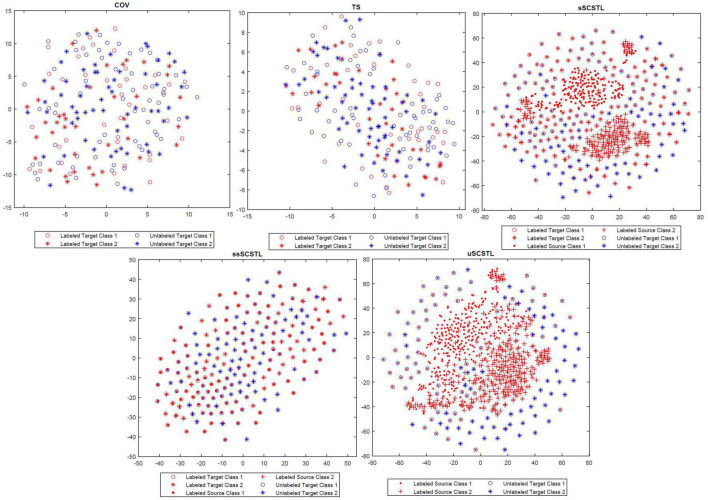
The feature distributions of different algorithms displayed in the two-dimensional space.

To appreciate the effect of cross-subject TL, in Figure 8, the t-SNE method was applied to visualize the feature distributions of the following Riemannian space-based and Riemannian tangent space-based algorithms, including COV, TS, sSCSTL, ssSCSTL, and uSCSTL (Van Der Maaten and Hinton, 2008). In our case, each feature was regarded as a point in a two-dimensional space. For the dataset MI1, the first 40 samples per class from good target subject 23 were labeled for training, the rest of samples were unlabeled for testing. Additionally, all samples from other good subjects were the labeled source samples.

In Figure 8, for COV and TS, the discrimination of two labeled target classes marked in red were obscure, resulting in the difficulty of classification for the unlabeled target samples marked in blue. However, after RA and TSM, the labeled target samples with same class for TS were closer than those for COV. sSCSTL and uSCSTL discriminated the two-class labeled samples from target and source subjects well, after source selection using the labeled and unlabeled target samples, respectively. Moreover, the classification performances of our proposed algorithms might increase since many unlabeled target samples overlapped with labeled source samples which had the same class. Although, ssSCSTL could not separate the two labeled classes well, it performed well in most cases.

### Computation Time Comparison

**FIGURE 9:**
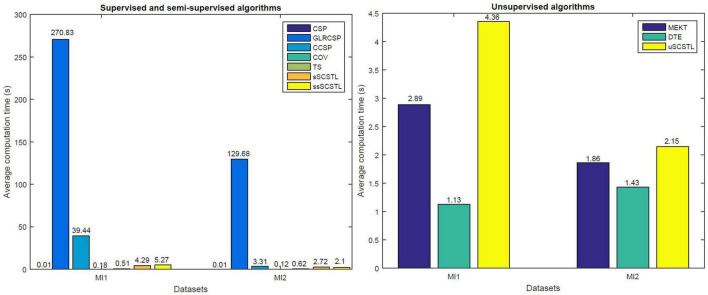
Computation time comparison of different algorithms.

Figure 9 shows average computation time of different algorithms on datasets MI1 and MI2. For supervised and semi-supervised algorithms, 40% of total samples from target subjects are used for training. For unsupervised algorithms, 60% of total samples from target subjects are unlabeled and used for cross-subject TL. Our experimental results were conducted in Matlab 2015a on a laptop with intel i5 CPU@1.60 GHz, 8 GB memory, running 64-bit Windows 10 Home Edition.

As shown in Figure 9, GLRCSP and CCSP spend much longer computation time than other algorithms when many good source subjects are available (seven for MI1 and five for MI2). Although CSP, COV, and TS save the running time, SCSTL and ssSCSTL spend acceptable running time. In addition, among all unsupervised algorithms, it takes the lowest time for DTE since DTE only selects one most appropriate source subject. The computation time of uSCSTL is also acceptable, although uSCSTL approximately spends twice running time of MEKT.

## Discussion

In this section, we discuss the experimental results from the following aspects.

(1) In terms of cross-subject TL

There are three non-TL algorithms (CSP, COV, and TS) in our paper. As shown in Figures 4-6, the average classification accuracies of TL algorithms are superior to those of non-TL algorithms in total. It proves the validity of cross-subject TL methods when few labeled samples from target subject are available, thus effectively shortening the calibration time for target subject. As depicted in Figure 9, compared with non-TL algorithms, most cross-subject TL algorithms spend more but acceptable running time except for GLRCSP and CCSP. However, the cross-subject TL algorithms in our paper are all offline algorithms, in which all samples from target and source subjects are ready in advance. Therefore, they are not suitable for real-time BCI applications.

(2) In terms of different feature spaces

The baseline algorithms and our proposed algorithms are divided into three categories according to their feature spaces, including Euclidean space-based, Riemannian space-based, and Riemannian tangent space-based algorithms. For Euclidean space-based cross-subject TL algorithms, GLRCSP and CCSP spend much time in computing optimal regularization parameters by means of cross-validation to weight the labeled samples from different subjects. For Riemannian tangent space-based algorithms, the hyper-parameters of MEKT and DTE are set by experience. Our proposed algorithms are parameter free. As shown in Figure 8, compared with Riemannian space-based algorithm COV, our proposed algorithms can make the samples from same class close to each other. In a word, our proposed algorithms can balance the classification performance and computation time well.

(3) In terms of different subjects

All subjects on MI1 or MI2 are separated into good and bad subjects according to their classification performances. To avoid negative transfer, most suitable source subjects are selected from good subjects for our proposed algorithms. As shown in Figures 4-7, our proposed algorithms exhibit obvious improvements over other algorithms for good target subjects. However, our proposed algorithms perform unsteadily for bad target subjects. The reason is that the classification performances of our proposed algorithms depend on the discriminability of the two-class target samples. For good target subjects, high discriminability of labeled or unlabeled target samples can help our proposed algorithms shorten the distances of samples with same class and select most appropriate source subjects. Nevertheless, for bad target subjects, low discriminability of labeled or unlabeled target samples influences the aggregation of the samples with same class and the source selection.

(4) In terms of labeled and unlabeled samples

To investigate the role of labeled and unlabeled samples, we propose sSCSTL, ssSCSTL, and uSCSTL. The labeled, total, or unlabeled samples from target subject are used to compute the Riemannian mean and source selection. As given in Tables 1, 5, with the help of unlabeled samples from target subject, ssSCSTL performs slightly better than sSCSTL. Possible explanation is that adding unlabeled samples from target subject can not only make all samples from target subject closer, but also be good for better source selection. As shown in Tables 2, 6, our proposed uSCSTL displays better classification performance than MEKT and DTE for good subjects. It suggests that unlabeled samples from good target subject which have inherent high discriminability can boost the performance improvement even if labeled samples from target subject are scarce.

(5) In terms of different classifiers

The baseline algorithms and our proposed algorithms use different supervised classifiers. Even for unsupervised TL algorithms, MEKT, DTE, and uSCSTL only use the unlabeled samples from target subject in the feature extraction phase. Except for TS, all Riemannian tangent space-based algorithms use sLDA as classifier. From the experimental results mentioned above, sLDA is much suitable for high-dimensional features. All Euclidean space-based algorithms and TS use LDA as classifier. As shown in Tables 1, 5, CSP outperforms TS in most cases. Possible explanation is that LDA may not cope with high-dimensional features of TS well. Although the MDM classifier is much suitable for the classification of covariance matrices, the COV algorithm does not perform ideally due to without RA.

## Conclusion

In this paper, we propose selective cross-subject transfer learning in supervised, semi-supervised, and unsupervised versions based on Riemannian tangent space. They perform RA using the available samples from target subject and the labeled samples from source subjects to preliminarily reduce the inter-subject differences. Then, all aligned covariance matrices from different subjects are converted into corresponding tangent space vectors for classification in Euclidean space. To realize positive transfer and relieve computational cost, the available tangent space vectors from target subjects are used to choose most suitable good source subjects based on SFFS. Experimental results show that our proposed algorithms are superior to several state-of-the-art algorithms, especially for good target subjects, when they have few or no labeled samples. Therefore, our proposed algorithms provide a new idea of solution for shortening calibration time in MI BCI.

However, our proposed ssSCSTL and uSCSTL algorithms are designed offline since the unlabeled samples from target subject are obtained a *prior*, instead of on-the-fly. Thus, we will further investigate how to adapt ssSCSTL and uSCSTl to the real-time BCI applications. Besides, ssSCSTL and uSCSTL only utilize the unlabeled samples from target subject in the feature extraction phase. Future work will be dedicated to extending ssSCSTL and uSCSTL in the classification phase to make full use of the unlabeled samples from target subject.

## Data Availability Statement

Publicly available datasets were analyzed in this study. This data can be found here: Dataset MI1: http://dx.doi.org/10.5524/100295, and Dataset MI2: http://www.bbci.de/competition/iv/desc_1.html.

## Author Contributions

YX wrote the manuscript, performed the research, and analyzed the experimental data. XH and QL designed, reviewed, and edited the manuscript. All authors read and approved the submitted manuscript.

## Conflict of Interest

The authors declare that the research was conducted in the absence of any commercial or financial relationships that could be construed as a potential conflict of interest.

## Publisher’s Note

All claims expressed in this article are solely those of the authors and do not necessarily represent those of their affiliated organizations, or those of the publisher, the editors and the reviewers. Any product that may be evaluated in this article, or claim that may be made by its manufacturer, is not guaranteed or endorsed by the publisher.
